# The Entomopathogenic Nematodes *H. bacteriophora* and *S. carpocapsae* Inhibit the Activation of proPO System of the Nipa Palm Hispid *Octodonta nipae* (Coleoptera: Chrysomelidae)

**DOI:** 10.3390/life12071019

**Published:** 2022-07-09

**Authors:** Nafiu Bala Sanda, Bofeng Hou, Youming Hou

**Affiliations:** 1 State Key Laboratory of Ecological Pest Control for Fujian and Taiwan Crops, Department of Plant Protection, Fujian Agriculture and Forestry University, 15, Shangxiadian Road, Cangshan District, Fuzhou 350002, China; nbsanda.crp@buk.edu.ng (N.B.S.); 2020202048@stu.njau.edu.cn (B.H.); 2 Department of Crop Protection, Faculty of Agriculture, Bayero University Kano, Gwarzo Road, Kano P.M.B. 3011, Nigeria; 3 Department of Entomology, College of Plant Protection, Nanjing Agricultural University, Nanjing 210095, China

**Keywords:** immune suppression, eicosanoid biosynthesis, entomopathogenic nematodes, prophenoloxidase activation, *Octodonta nipae*

## Abstract

Entomopathogenic nematodes are biocontrol agents of invasive insect pests in soil and cryptic habitats. Nipa palm hispid, *Octodonta nipae*, is a pest of palm trees in Sothern China. To address its increasing damage, environmentally friendly control methods are required. This study aimed to test efficacy of *Heterorhabditis bacteriophora* and *Steinernema carpocapsae* on *O. nipae* and investigated the influence of secondary metabolites, nematodes, and their isolated cuticles on the activation of *O. nipae*’s prophenoloxidase system using qPCR analysis. Our data revealed that *O. nipae* were less susceptible to *H. bacteriophora* than *S. carpocapsae* and penetrations of infective juveniles were higher with *S. carpocapsae* treatment than *H. bacteriophora*. Moreover, expression levels of the serine protease P56, prophenoloxidase activation factor 1, *PPO* and serine protease inhibitor 28 upon *S. carpocapsae* and *H. bacteriophora* infections were generally downregulated at all times. However, upon heating, the cuticles lost their inhibitory effects and resulted in upregulation of the *PPO* gene. Similarly, the addition of arachidonic acid reversed the process and resulted in the upregulation of the *PPO* gene compared to the control. Further work is needed to identify toxic substances secreted by these EPNs to evade *O. nipae*’s immune system.

## 1. Introduction

The movement of destructive pest species from one country or region to another is on the rise. Nipa palm hispid *Octodonta nipae* (Maulik) is not an exception [1,2,3,4,5,6,7,8,9]. This pest is one of the most destructive invasive insects, reportedly causing economic damage to palm trees in China, mainly in Hainan, Guangdong, Fujian, etc. [9,10,11,12,13,14,15,16]. However, due to hazard caused by insecticides to the environment, the use of chemical control of this pest remains elusive. Several attempts were made by different studies to control this beetle using non-chemical methods [17,18,19]. One of these was the use of *Tetrastichus brontispae* Ferrière (Hymenoptera: Eulophidae) [20,21,22,23,24,25,26,27,28] entomopathogenic nematode *Steinernema carpocapsae* [17,18,19] and *Metarrhizium anisopliae* [29,30,31,32]. More eco-friendly and cost-effective methods of control of *O. nipae* are needed.

Heterorhabditidae and Steinernematidae are families of entomopathogenic nematodes (EPNs) mainly used for biological control of different economically invasive pests at field and laboratory levels [33,34,35,36,37]. They have advantages over the conventional chemical control because they have the ability to actively search for their hosts in cryptic habitats like leaf surfaces [38,39,40,41]. In addition, EPNs are known to be more effective against the larvae and other pre-adult stages of insects because they enter the host body easily [42]. EPNs are reported to be used for the control of leaf-eating caterpillars and other insect pests of many crops [43]. Many researchers have studied the efficacy of EPNs in controlling the adult western corn rootworm, *Diabrotica virgifera* LeConte [37,44], flea beetles, *Phyllotreta* spp. [45], and Colorado potato beetle, *Leptinotarsa decemlineata* (Say) found on leaf surfaces [40]. Similarly, the use of EPN for the control of *O. nipae* larvae was conducted in our laboratory using *S. carpocapsae* and found to be virulent at different concentrations and time points, as published in Sanda et al. [19]. However, experiments to test the efficacy of Heterorhabditis bacteriophora on *O. nipae* larvae have yet to be conducted.

Insects employ both humeral and cellular immune reactions in response to attacks by pathogens such as bacteria, fungi, and nematodes. These innate immune mechanisms include nodulation, phagocytosis, encapsulations, production of antimicrobial peptides, and activation of PPO system, which leads to melanization and subsequent pathogen death by septicemia [46,47]. Insect PPO activation system is one of the immune reactions against nematodes and bacteria that involves several proteinases, which are regulated by specific inhibitors. When pathogens like nematodes or bacteria attack insects, the hemolymph pattern-recognition proteins such as Peptidoglycan recognition proteins (PGRP) and C-type lectin bind the pathogens’ surface polysaccharides, thereby inducing the activation of initiator protease(s). In addition, enzymes released by the pathogens, as well as tissue damage due to the infection, may activate the proPO system [48,49]. These proteases trigger a protease cascade, activating a series of terminal serine proteases such as proPO-activating enzymes (PPAE), proPO-activating proteinase (PAP), or proPO-activating factors (PPAF) to cleave PPO and form active phenoloxidase (PO). However, the excessive production of these proteases is regulated by serine proteinase inhibitors (serpins) that limit the reaction speed and avoid excessive melanization [49,50]. In *O. nipae*, three full-length cDNAs of PPAFs (OnPPAFs) were cloned, namely OnPPAF3, OnPPAF1, and OnPPAF2. They were highly expressed in hemolymph except OnPPAF2, which shows low transcript abundance. However, knockdown of OnPPAF1 and OnPPAF3 showed a reduction in hemolymph phenoloxidase activity and inhibition of hemolymph melanization [51,52,53,54].

Moreover, different insect hosts responded differently to EPNs infections depending on the species and strain. Each nematode species uses different strategies to evade and suppress melanization and escape cellular encapsulation. Our previous study showed that EPNs of the family Steinernematids, *S. carpocapsae* use their body surface cuticle to escape host hemocyte encapsulation and at the same time inhibit its proPO system activity [19]. These are believed to be triggered by the body surface of the Steinernematids due to the presence of some lipid compounds on its body cuticle. Additionally, pathogenicity of nematodes is believed to be essentially modulated by the nematode’s excreted and secreted products (ESPs), which are proteases and protease inhibitors that act against effectors of the host’s immune system [55]. These ESPs have been detected in *S. carpocapsae* and are involved in host immune modulation [23,56,57].

Eicosanoids are other insect cellular and humoral immune mediators against several microbial infections [58,59]. They are mainly synthesized from arachidonic acid (AA) (5,8,11,14-eicosatetraenoic acid). AA is linked to phospholipids at the sn2 position and subsequently released upon microbial challenge by the catalytic activity of phospholipase A2 (PLA2) [60]. Biosynthesis of eicosanoids could also be inhibited by a nematode–bacteria complex [61]. Inhibition of PLA2 due to suppression of eicosanoids results in subsequent inhibition of phenoloxidase activation [62]. The symbiotic bacteria produce secondary metabolites such as Oxindole, Dexamethasone (DEX), and Benzylideneacetone (BZA) that suppress the activity of PLA2, thereby inhibiting the host immune responses [63,64]. These metabolites also restrict the spread of hemocytes and inhibit nodulation, antimicrobial peptides (AMPs) expression, and phenoloxidase activity [65,66,67]. However, the molecular nature of how *H. bacteriophora* and *S. carpocapsae* interact with *O. nipae* humoral immune system remains unknown. The present study, therefore, was conducted to determine the survival rates of *O. nipae* larvae at different concentrations of *H. bacteriophora*. Similarly, we evaluated the penetration abilities of both H. bacteriophora and *S. carpocapsae* against the third instar larvae of *O. nipae*. Further, we tested the differences in expression levels of four selected genes involved in proPO activation in *O. nipae* larvae. Finally, the effects of some selected secondary metabolites and isolated cuticles from nematodes on *O. nipae* proPO gene expression were investigated.

## 2. Materials and Methods

### 2.1. Experimental Samples

*H. bacteriophora* H06 (Poinar) and *S. carpocapsae* All (Weiser) were obtained from the Guangdong Institute of Applied Biological Resources, China [68]. Nematodes were cultured using larvae of greater wax moth, *Galleria mellonella* [69]. Infective Juveniles (IJs) were stored in distilled water at 15 °C and were used in all experiments within 15 days of emergence from the host. Before the experiments, nematodes were kept at 25 °C for 60 min [70]. *O. nipae* adults were collected from Hainan Island, China, and reared with small pieces of fortunes windmill palm, *Trachycarpus fortunei* (Hook) in the laboratory for many generations, after which healthy third instar larvae were selected and used for this study. The insects were maintained at 25 ± 1 °C, relative humidity of 80 ± 5%, and a photoperiod of 12 light: 12 dark hours, as previously described by Sanda et al. [18].

### 2.2. Reagents

The phospholipase A_2_ inhibitor, dexamethasone (DEX: (11β,16α)-9-fluoro-11,17,21-trihydroxy-16-methylpregna-1,4-diene-3), the eicosanoid precursor, arachidonic acid (AA: 5,8,11,14-eicosatetraenoic acid), serine proteases inhibitor, phenylmethanesulfonyl (PMSF) fluoride, and dimethyl sulfoxide (DMSO) were purchased from Sigma Aldrich (Shanghai, China) Trading Co., Ltd. (Sigma Aldrich Research Biochemicals, Pudong District, Shanghai, China).

### 2.3. Virulence of Entomopathogenic Nematodes on O. nipae Larvae

Here, the effects of EPN concentrations on mortality of *O. nipae* larvae were explored. In addition, the ability of the IJs to penetrate the *O. nipae* larvae at 0, 25, 50 and 100 IJs/larva concentrations was also experimented, and complete randomized design (CRD) was used as experimental design. Bioassay was conducted to determine the survival of *O. nipae* larvae at different concentrations of *H. bacteriophora* as fully described in our previous experiment using *S. carpocapsae* [18]. In the same vein, bioassay was conducted to determine the IJs penetration of both *H. bacteriophora* and *S. carpocapsae*. After 72 h of inoculation, ten dead larvae (cadavers) were picked from the well plates, rinsed with distilled water, and dissected under dissecting stereo-microscope (Nikon SMZ745T Stereo, Camera; Nikon DS-fi2)-Nikon company, Japan. The number of penetrated IJs were counted and recorded. Distilled water was used as control and each treatment contained 30 larvae, replicated three times to confirm the results.

### 2.4. Expression of Four Selected Prophenoloxidase Activation Genes in O. nipae Post-Nematodes Infections

We intended to determine the effects of EPN infections on the mRNA expression level of four selected genes involved in the activation of the proPO system of *O. nipae* larvae infected with the two nematode species. These include Serine Protease P56 (*SPP56*), prophenoloxidase activation factor 1 (*PPAF1*), *PPO*, and serine protease inhibitor 28 (*SPI28*) genes (Table 1). Third instar larvae of *O. nipae* were infected with *H. bacteriophora* and *S. carpocapsae* at 100 IJS per larva, as described above. Larvae samples were collected from each treatment at 8, 16, and 24 h after inoculation. Total RNA isolation, cDNA Synthesis, and qRT-PCR analysis and calculations were performed as described in Sanda et al. [19]. Ribosomal protein S3 (rpS3) was use as a reference gene, and qRT-PCR analysis was performed in triplicate for each biological replicate.

### 2.5. Influence of Nematodes Isolated Cuticle on the Expression Analysis of PPO Gene

To determine whether nematode body cuticle have an influence on immune expression of *O. nipae PPO gene*, *H. bacteriophora* and *S. carpocapsae* body cuticles were isolated and purified as described in our previous study [19]. The *O. nipae* larvae were injected with 112 nL of purified isolated cuticles from *H. bacteriophora* and *S. carpocapsae* and the same amount of phosphate buffered saline (PBS) as control. Another set of the cuticles were subjected to heat treatment at 100 °C for 20 min. The heat-treated cuticles were injected in the same way as mentioned above. Total RNA isolation and qRT-PCR analysis were performed in triplicate for each biological replicate according to Sanda et al. [19] to check the *PPO* gene expression levels due to the untreated cuticle, heat-treated cuticle, and control treatments.

### 2.6. Influence of Dexamethasone (DEX) and Arachidonic Acid (AA) on PO Activity in O. nipae

We determined the inhibition effects of Dexamethasone’s presence on the phenoloxidase activity in an in vitro assay with hemolymph of *O. nipae* larvae, as described in our previous study [19]. Secondly, AA was added on inhibitors’ treatments to reverse the inhibition effects caused by dexamethasone. All the kinetics were performed as described in Sanda et al. [19]. For control treatment, only 20 µL of distilled water was added in place of various treatments. The relative activity of phenoloxidase was measured with a spectrophotometer (SpectraMax, Molecular Devices Corporation, San Jose, CA, USA) at 490 nm 5 min^−1^, and at 20 °C. The experiments were run in triplicate to confirm the results.

### 2.7. Combine Effects of Dexamethasone (DEX), Arachidonic Acid (AA), and Nematodes Treatments on the Expression Level of PPO Gene

We injected the *O. nipae* larvae with eicosanoid biosynthesis inhibitor, dexamethasone (DEX) and precursor, arachidonic acid (AA) individually and in combination with *H. bacteriophora* and *S. carpocapsae* to ascertain their roles on the expression of *PPO gene*. The inhibitor, DEX, was dissolved in 100% dimethyl sulfoxide (DMSO) at an initial concentration of 1 M and subsequently diluted to 5 mM concentration. Three treatment groups were prepared for injection into the *O. nipae* larvae at 112 nL using a Nanoliter 2010 injection system (World Precision Instruments, Sarasota, FL, USA). The first group was injected with DEX only to test its inhibitory effects on *O. nipae* proPO activation system, and PBS was used as control. The second group of the larvae was initially infected with nematodes and then injected with DEX after 8 h post-infection. The last group was infected with nematodes + AA to recover the effects of nematode inhibition. Samples were taken at 24 h after treatment for RNA isolation. Total RNA isolation and qRT-PCR analysis were performed in triplicate for each biological replicate according to Sanda et al. [19] to investigate the *PPO* gene expression levels of each group.

### 2.8. Statistical Analyses

All statistical analyses were performed using IBM SPSS Statistics version 22 (IBM Corporation, New York, USA) (SPSS, RRID: SCR_002865). Data were corrected for control mortality using Abbott formula [34] and percentage data were square-root transformed to meet the assumptions of normality and homogeneity of variances. The percentage-corrected cumulative mortality data, penetration data, were analyzed by one way analysis of variance (ANOVA). When ANOVA showed a significance effect (*p* ˂ 0.05), means were compared or separated using least significance differences (LSD). The level of mRNA expression of some selected genes in proPO activation system of *O. nipae* was transformed by Logarithmic function and analyzed using the Student’s *t*-test. Differences between mean values were analyzed and considered significant when *p* < 0.05 or considered extremely significant when *p* < 0.0001 concerned the control values.

## 3. Results

### 3.1. Survival of O. nipae Larvae Infected with H. bacteriophora and S. carpocapsae

**Figure 1:**
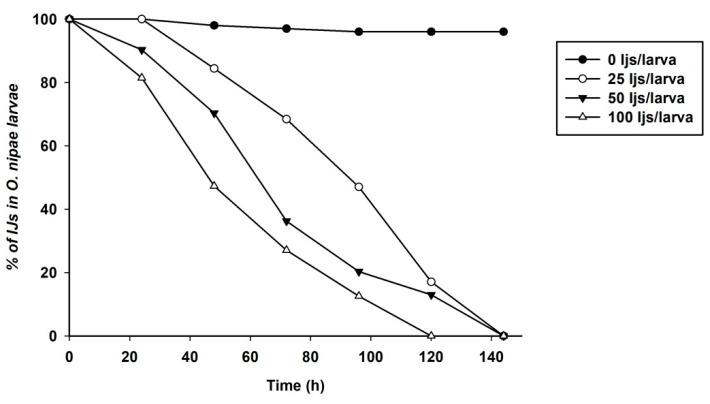
Survival curve of *O. nipae* larvae infected with *H. bacteriophora* at different concentrations are significantly different, (*p* < 0.0001, log-rank test).

We previously evaluated the pathogenicity of *S. carpocapsae* at different concentrations by plotting the survival curve at different time points [19]. Significant differences in survival of *O. nipae* larvae at different concentrations were reported and at 24 and 48 h post-treatments [19]. In this study, the pathogenicity of *H. bacteriophora* on the survival of *O. nipae* larvae was evaluated. The results showed that the survival ability of *O. nipae* larvae was significant at different time points and *H. bacteriophora* IJs concentrations (*χ*^2^ = 42.170, df = 4, *p* = 0.001, Log-Rank Test). The *O. nipae* larvae survived for 140 h longer at lower concentrations of 25 and 50 IJs/larva than at 100 IJs/larvae, as shown in Figure 1.

**Figure 2:**
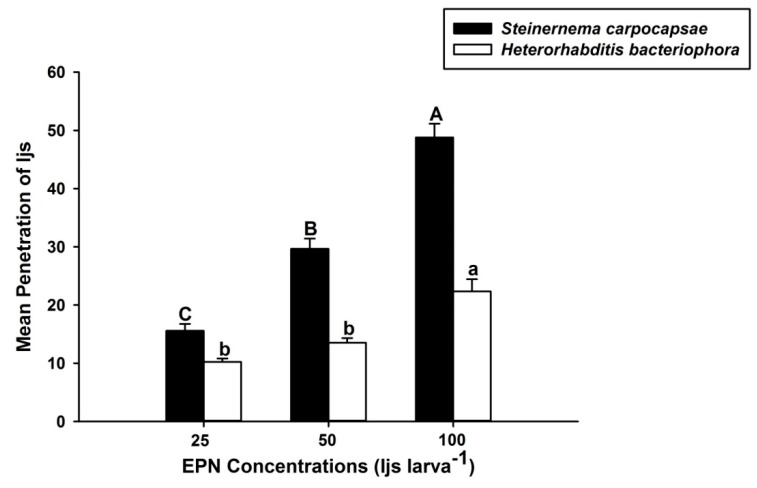
Mean penetration of infective juveniles of *Heterorhabditis bacteriophora* and *Steinernema carpocapsae* at different concentrations into third instars of *Octodonta nipae*. Means with the same letter are not significantly different, capital letters represent means of *S. carpocapsae* penetration, lowercase letters represent means of *H. bacteriophora* penetration.

Similarly, the results for the penetration assay revealed that the IJs of *S. carpocapsae* and *H. bacteriophora* penetrated the hemocoel of the larvae of *O. nipae* at the concentration of 25, 50, and 100 IJs/larva (Figure 2). However, the mean penetration potential of *S. carpocapsae* was significantly higher (*F*_2,6_ = 60.84, *p* < 0.01) than that of *H. bacteriophora* (*F*_2,6_ = 23.99, *p* = 0.01) in *O. nipae* larvae, as shown in Figure 2. The mean penetration of IJs increased with increasing IJs concentration, in both *S. carpocapsae* and *H. bacteriophora*.

### 3.2. S. carpocapsae and H. bacteriophora Infection Suppress the Activation of O. nipae PPO System

**Figure 3:**
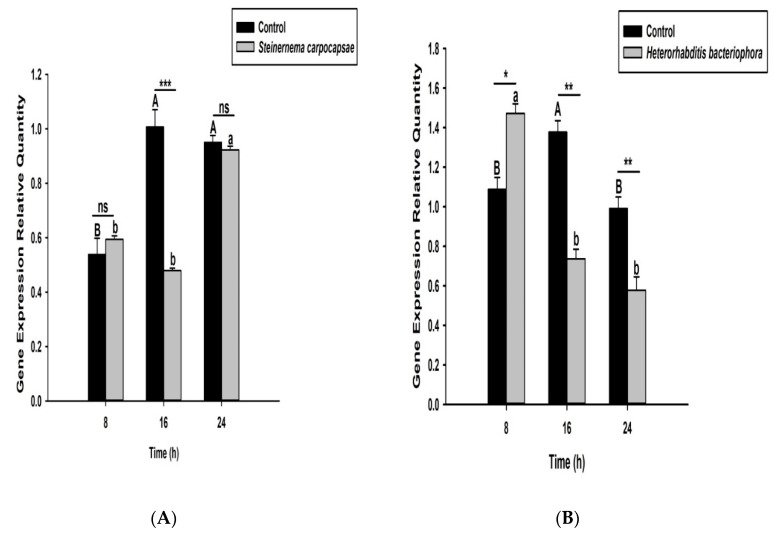
Transcription of Serine Protease P56 (*SPP56*) gene in *O. nipae* larvae infected with (**A**) *S. carpocapsae* and (**B**) *H*. *bacteriophora* at 8, 16, and 24 h after treatments. Error bars labeled with different letters are significantly different (one-way ANOVA followed by LSD test, *p* < 0.05). The asterisks *** (*p* < 0.0001); ** (*p* < 0.001); * (*p* < 0.01) indicates different significant levels between the control and nematode treatments at the indicated time period, while “ns” indicates no significant difference.

**Figure 4:**
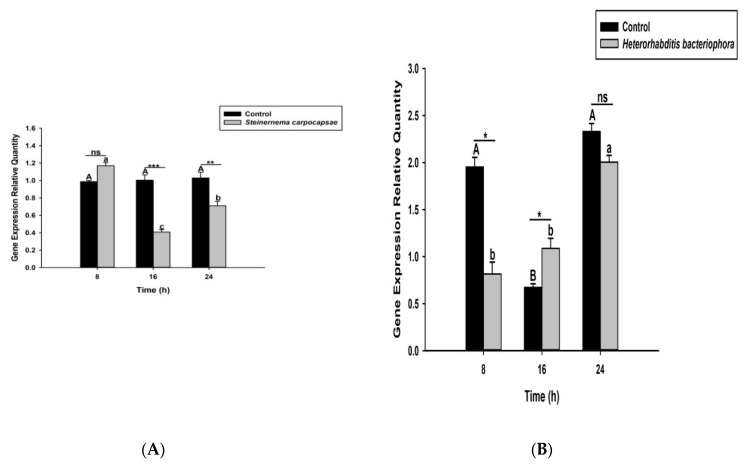
Transcription of prophenoloxidase Activation Factor 1 (PPAF1) gene in *O. nipae* larvae infected with (**A**) *S. carpocapsae* and (**B**) *H*. *bacteriophora* at 8, 16, and 24 h after treatments. Error bars labeled with different letters are significantly different (one-way ANOVA followed by LSD test, *p* < 0.05). The asterisks *** (*p* < 0.0001); ** (*p* < 0.001); * (*p* < 0.01) indicate different significant levels between the control and nematode treatments at the indicated time period, while “ns” indicates no significant difference.

**Figure 5:**
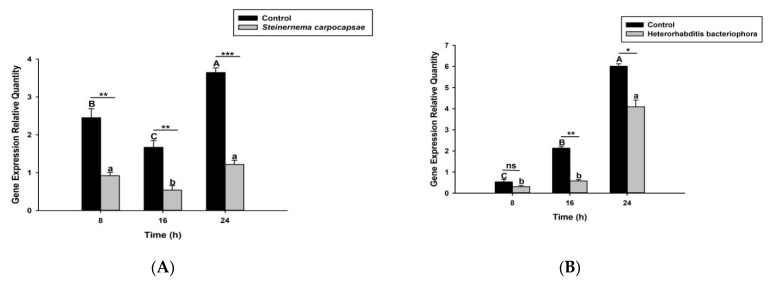
Transcription of prophenoloxidase (*PPO*) gene in *O. nipae* larvae infected with (**A**) *S. carpocapsae* and (**B**) *H*. *bacteriophora* at 8, 16, and 24 h after treatments. Error bars labeled with different letters are significantly different (one-way ANOVA followed by LSD test, *p* < 0.05). The asterisks *** (*p* < 0.0001); ** (*p* < 0.001); * (*p* < 0.01) indicate different significant levels between the control and nematode treatments at the indicated time period, while “ns” indicates no significant difference.

**Figure 6:**
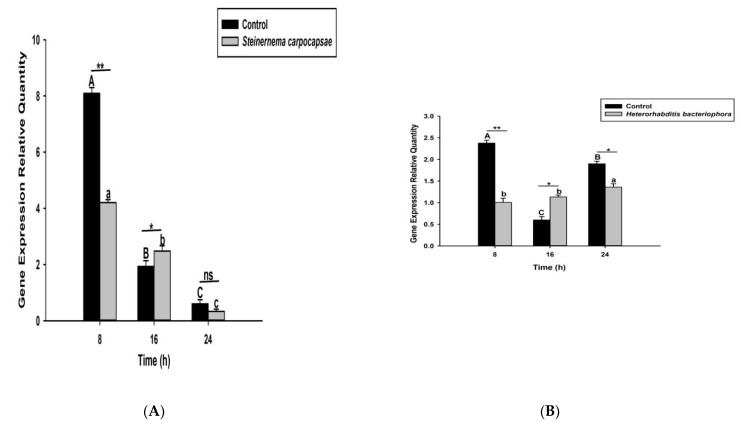
Transcription of serine protease inhibitor 28 (SPI28) gene in *O. nipae* larvae infected with (**A**) *S. carpocapsae* and (**B**) *H*. *bacteriophora* at 8, 16, and 24 h after treatments. Error bars labeled with different letters are significantly different (one-way ANOVA followed by LSD test, *p* < 0.05). The asterisks; ** (*p* < 0.001); * (*p* < 0.01) indicate different significant levels between the control and nematode treatments at the indicated time period, while “ns” indicates no significant difference.

Here we investigated and compared the expression levels of four *O. nipae* prophenoloxidase enzymes (including the Serine Protease P56, *SPP56*; prophenoloxidase activation factor 1, *PPAF1*; *PPO*; and serine protease inhibitor 28, *SPI28*) between *H. bacteriophora*- and *S. carpocapsae*-treated larvae at three distinct time intervals. Our qRT-PCR results reveals that *SPP56* was significantly upregulated (*t*_4_ = 1.346, *p* = 0.022) after *H. bacteriophora* challenge and insignificantly upregulated (*t*_4_ = 2.93, *p* = 0.383) upon *S. carpocapsae* treatment at 8 h post-infection (Figure 3A,B). At 16 and 24 h post-treatment, however, *SPP56* was downregulated upon a challenge with both nematodes (Figure 3A,B). These translate to differences in the invasion strategies between the two nematodes. *PPAF1* was downregulated at all time points following treatment with both nematodes, except at 16 h where *H. bacteriophora* infection results in significant upregulation (*t*_4_ = 1.88, *p* = 0.047) of the gene (Figure 4A,B). Similarly, the mRNA expression levels of *PPO* gene were completely downregulated at 8, 16, and 24 h post-*S. carpocapsae* and *H. bacteriophora* treatments (Figure 5A,B). As expected, the serine protease inhibitor *SIP28* was downregulated in all nematodes treatments at 8 and 24 h time points, except at 16 h where it appeared upregulated for *S. carpocapsae* (*t*_4_ = 1.31, *p* = 0.032) and *H. bacteriophora* (*t*_4_ = 1.58, *p* = 0.040) treatments (Figure 6A,B). To sum it up, the *S. carpocapsae* and *H. bacteriophora* treatments successfully inhibit the *PPO* activation system of *O. nipae*, except at a few time-points where it shows upregulation.

### 3.3. The Heat-Treated Cuticles Positively Expresses the PPO Gene in O. nipae

**Figure 7:**
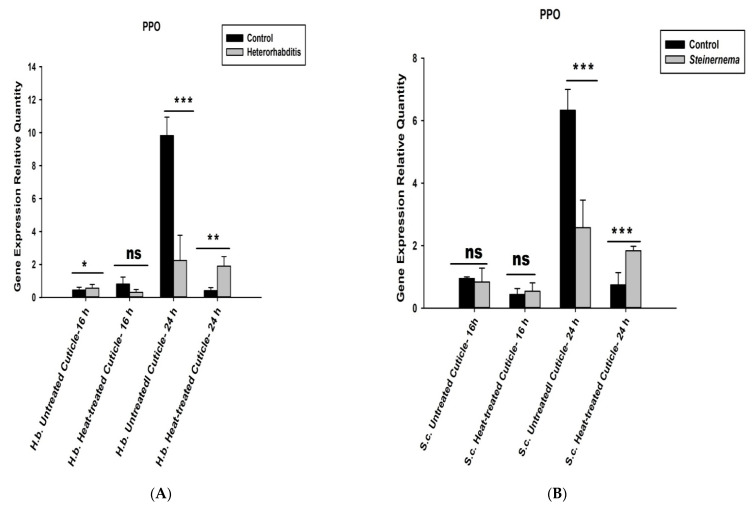
Transcription of prophenoloxidase (*PPO*) gene in *O. nipae* larvae injected with (**A**) *H. bacteriophora* purified cuticle (**B**) *S. carpocapsae* purified cuticle. Treatments with heat-killed cuticles reversed the influence of untreated cuticles on the PPO expressions. H.b.—*H. bacteriophora* and S.c.—*S. carpocapsae*. The asterisks *** (*p* < 0.0001); ** (*p* < 0.001); * (*p* < 0.01) indicates different significant levels between the control and nematode treatments at the indicated time period, while “ns” indicates no significant difference.

The expression level of *O. nipae* *PPO* gene challenged with nematodes isolated cuticles was generally low compared to the control treatment. The mRNA level of the *PPO* gene was insignificantly regulated at 16 h (*t*_2_ = 1.28, *p* = 0.272 ns) but significantly downregulated at 24 h (*t*_2_ = 1.817, *p* = 0.001) after injection of the isolated cuticles from the two nematodes (Figure 7). To further confirm this, we subjected the isolated cuticles to heat treatment at 100 °C for 20 min and injected them into the larvae as described above. The results establish that the expression levels of *PPO* gene are higher than the controls, especially at late hours after injection in both treatments. A significant increase in mRNA level of *PPO* gene was obtained at 24 h post-*H. bacteriophora* (*t*_2_ = 2.93, *p* = 0.030) and *S. carpocapsae* (*t*_2_ = 1.31, *p* = 0.001) heat-treated cuticles injections. At 16 h after injection of *S. carpocapsae* cuticle, insignificance (*t*_2_ = 1.27, *p* = 0.152) upregulation of *PPO* gene was recorded (Figure 7A,B).

### 3.4. Addition of Arachidonic Acid Reverses the Inhibitions of Phenoloxidase Activity Caused by Dexamethasone

**Figure 8:**
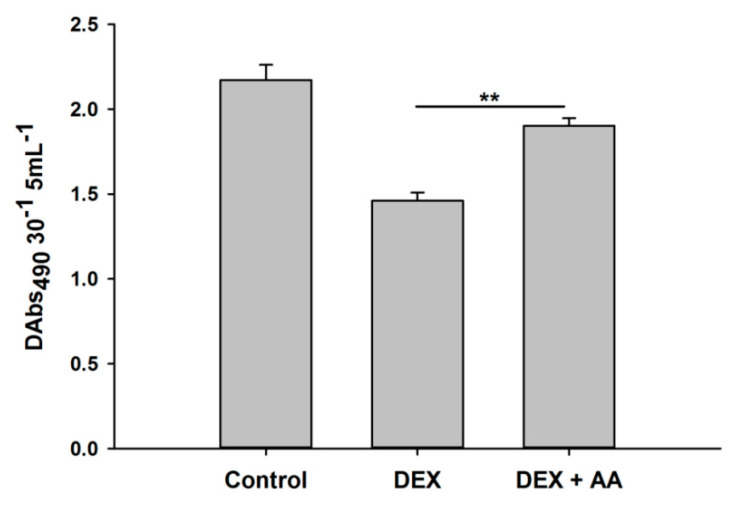
Effects of Dexamethasone (DEX) and Oxindole on inhibition of phenoloxidase (PO) activity in third instar *O. nipae* larvae. The addition of Arachidonic acid (AA) reverses the inhibition of PO activity caused by metabolite treatments. The asterisks ** (*p* < 0.001) indicate highly significant levels between the control and treatments.

In this study, phenoloxidase-inhibitory activities of eicosanoid biosynthesis inhibitors and DEX treatments compared to controls were observed. There were significant decreases in phenoloxidase enzyme activities in hemolymph of *O. nipae* larvae treated with DEX (*F*_2,6_ = 42.13, *p* = 0.002). However, the addition of AA rescued the phenoloxidase-inhibitory activities caused by DEX treatments (Figure 8). There were significant increases in phenoloxidase activities in hemolymph of *O. nipae* larvae treated with DEX +AA (*F*_2,6_ = 43.13, *p* = 0.001).

### 3.5. Treatment with DEX Suppresses the Expression of the PPO Gene in O. nipae

**Figure 9:**
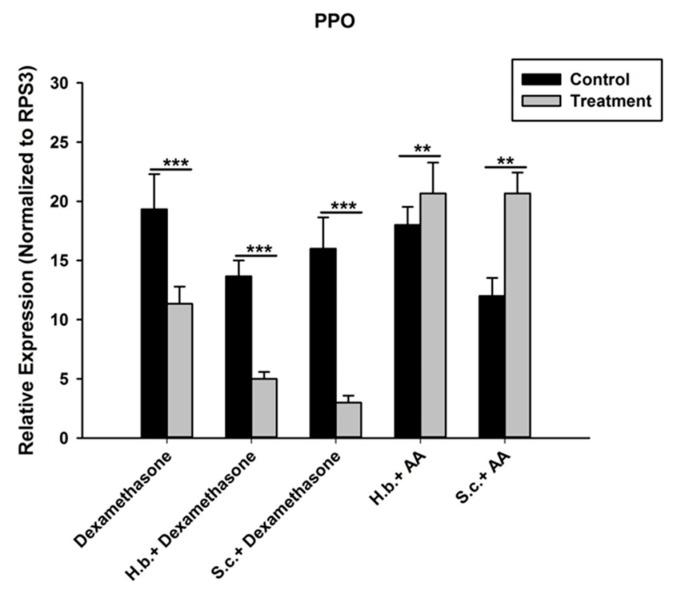
Influence of DEX and nematodes plus DEX, on expression of prophenoloxidase (*PPO*) gene in *O. nipae* larvae. AA rescues DEX and nematodes plus DEX-suppressed expression of *PPO* gene in *O. nipae* larvae. The asterisks *** (*p* < 0.0001); ** (*p* < 0.001); indicate different significant levels between the control and nematode treatments at the indicated time period.

To further ascertain the role of this eicosanoid biosynthesis inhibitor DEX, we determined the expression level of *PPO* gene in treated larvae samples. Our results revealed that the expression level of *PPO* gene was downregulated significantly upon treatment of DEX (*t*_4_ = 4.20, *p* = 0.001) compared with control (Figure 9).

Secondly, injections of DEX to nematodes-treated larvae further suppressed the expression level of *PPO* gene. There were significant further downregulations of *PPO* gene in *H. bacteriophora* plus DEX (*t*_4_ = 2.928, *p* = 0.001) and *S. carpocapsae* plus DEX (*t*_4_ = 1.91, *p* = 0.002) treatments at 24 h. Interestingly, injection of AA (precursor of eicosanoid biosynthesis) to nematode-treated larvae reverses the inhibition effects of both DEX and nematode plus DEX-treated larvae on the *PPO* gene expression. The mRNA level of *PPO* gene in *H. bacteriophora* plus AA (*t*_4_ = 1.346, *p* = 0.024) and *S. carpocapsae* plus AA (*t*_4_ = 2.93, *p* = 0.030) treatment were highly significant at 24 h after treatments.

## 4. Discussion

Entomopathogenic nematodes modulate their hosts differently, depending on the strategies used to escape the host defense mechanisms. For example, Zang and Maizels [55] described the pathogenicity of insect pathogenic nematodes against their target hosts as resulting from an “arms race” between the insect and the nematodes. The findings from this study suggested that *O. nipae* larvae were more susceptible to *H. bacteriophora*. Compared to our previous results, the virulence of *S. carpocapsae* [19] over *H. bacteriophora* can be attributed to their differences in host-searching strategies, as reported by Grewal et al. [38] and Koppenhöfer et al. [71]. Additionally, insect cellular immune responses differ among species and depend on the invading species. For instance, *G. mellonella* hemocytes can recognize *H. bacteriophora* IJs, but not *S. carpocapsae* and *S. glaseri* nematodes [72]. This is similar to our previous experiment where *S. carpocapsae* efficiently resists being encapsulated and melanized within the *O. nipae*’s hemolymph and most of the nematodes were observed moving freely in the hemolymph even at 24 h post-incubation, whereas *H. bacteriophora* was identified, encapsulated and subsequently melanized by *O. nipae*’s hemolymph [19]. Moreover, Memari et al. [73] reported that *H. bacteriophora* was less virulent to carob moth *Ectomyelois ceratoniae* Zeller (LC_50_ = 426.9 IJs larva^−1^) when compared to both *S. carpocapsae* and *S. feltiae* (LC_50_ = 2 IJs/larva) at concentrations similar to this study. Additionally, Sharifi et al. [74] have reported that *S. carpocapsae* caused higher mortality of *Osphranteria coerulescens* than *H. bacteriophora*, as evident by their LC_50_ of 2.7 and 9.0 IJs larva^−1^, respectively. This is consistent with quite a number of reports for different insect pest control [75,76,77,78,79,80].

In any successful biological control program using EPNs as control agents, the nematodes should have the ability to not only penetrate their hosts but also reproduce inside them. The routes of entry of IJs into the host body cavity differ among nematode species and insect hosts. These require some mechanical processes by the nematodes to bypass the insects’ natural barriers, such as hairs [81]. According to Sharifi et al. [74], different hosts and EPN species have different penetration rates. In this study, we reported that differences exist between concentrations of *S. carpocapsae* and *H. bacteriophora* with regards to the mean penetration potential within the host larvae. This is also similar to a report by Susurluk et al. [82], which established higher penetration rates of *S. carpocapsae* into turf pest, *Dorcadion pseudopreissi*, and lower in *H. bacteriophora* treatments. Similarly, higher penetration rates were also reported for Steinernematids in previous works by Phan et al. [83], Salari et al. [34], Salem et al. [84], Koppenhöfer et al. [71], and Caroli et al. [85].

During host infection, nematodes contend the humoral and cellular host immune responses through passive and active strategies, in which nematodes avoid triggering an immune response by being recognised as ‘*self*’ as in *S. carpocapsae*. They also suppress the hosts’ immune responses after being recognised as ‘*non-self*’ [86,87]. In addition, nematodes modulate their hosts’ immune responses by releasing toxic molecules (such as proteases and inhibitors) during infection [88,89,90]. Activation of proPO results in the synthesis of phenoloxidase (PO), which serves as a reactive intermediate for melanin biosynthesis, an important component of insect’s innate immunity which can be activated by serine proteinases [55]. However, some EPNs have developed some means of preventing proPO activation by producing serpin-type inhibitors or other factors specifically interfering with the proteolytic activation of proPO or its upstream components. Here, we dissected the expression levels of four proPO activation enzymes of *O. nipae* infected with *S. carpocapsae* and *H. bacteriophora*. From our results, we found that nearly all the genes involved were downregulated in both nematodes and at all time points, except in a few cases. Initially, when the serine protease *SPP56* was majorly downregulated, the whole enzymes involved in the activation of proPO cascades are shut down. This is consistent with our previous study where *O. nipae*’s phenoloxidase activity was found to be slightly suppressed due to infection of both *H. bacteriophora* and *S. carpocapsae* at early stages of infection [19].

Additionally, the *S. carpocapsae* isolated cuticles escaped being melanized by *O. nipae*’s hemolymph in vitro and were found to be completely melanized, however, when isolated cuticles were heat-killed before treatment. This is in line with some previous reports that show success in parasitization of Steinernematids is aided by their body surface cuticles [89,90,91,92,93]. Similarly, *S. feltiae* was shown to prevent the antibacterial immune response in *G. mellonella* by their body surface [94]. Akhurst and Boemare [40] suggest that cuticles of parasitic nematodes in addition to secreted molecules are involved in immune-evasion and suppression of host’s defences during the invasion. It was also reported to depress the host immune system, such as the proPO system, when injected into the hemocoel. Therefore, it was hypothesized that the nematode disguises as ‘self’ with the hemolymph proteins of the invading host by coating its body surface [94]. Yi et al. [59] showed that the involvement of *S. carpocapsae* or *H. bacteriophora* cuticles caused suppressions of humoral and cellular immune processes, such as phenoloxidase activity, haemocytes vitality, phagocytosis and antimicrobial activity. In this study, we confirm that the purified, isolated cuticles suppress the expression level of *PPO* gene in both nematodes’ treatments. However, upon heating, the cuticles lost their inhibitory effects on *O. nipae*’s immune responses and resulted in the upregulation of the *PPO* gene at both 16 and 24 h after treatment.

Furthermore, eicosanoids mediate basic humoral and cellular immune response mechanisms in insects [89]. These include nodulation, phagocytosis, encapsulation, and phenoloxidase activation systems. The results of the present experiment revealed that the eicosanoid biosynthesis inhibitor DEX suppresses the expression of the *PPO* gene in *O. nipae*, similar to that caused by treatment with *H. bacteriophora* and *S. carpocapsae*. In contrast, the addition of AA reversed the process and resulted in the upregulation of the PPO gene compared to the control treatment. In a study by Yi et al. [59], injection of the eicosanoid biosynthesis inhibitors dexamethasone and indomethacin induced similar depression in the key innate immune parameters, such as PO activity, compared to the nematode cuticles treatment in *G. mellonella*. However, injection of larvae with AA reverses the inhibitory effects of these inhibitors. Similarly, benzaldehyde and its derivative treatments resulted in the inhibition of PO activity in *Pieris rapae* larvae [95]. This is supported by the study of Ullah et al. [96] in which, in addition to the inhibition of nodule formation, the PO activity was also inhibited at different concentrations of benzaldehyde in *G. mellonella* larvae. Similarly, treatments with BZA (benzylideneacetone) in another study resulted in the immunosuppression of PO activities in *Plutella xylostella* and *S. exigua* [97,98,99]. We conclude that various eicosanoid inhibitors play a crucial role in the death of target insects.

## 5. Conclusions

Overall, this study provides the first data on the use of *H. bacteriophora* and *S. carpocapsae* for biological control of *O. nipae* larvae at different concentrations in the laboratory. We further dissected the expression levels of four selected proPO activation enzymes infected with *H. bacteriophora* and *S. carpocapsae* and found out that all of the genes were downregulated from both nematodes’ treatments. These were further confirmed by the counter experiments of heat-treated cuticles and AA treatment, which reversed the inhibitory effects caused by the nematodes and the eicosanoid inhibitor. Therefore, we speculated that these nematodes devised some means to evade and suppress the immune system of *O. nipae*, either by secretion of toxins or by manipulation of the host system. Future work will focus on the identification and functional analysis of the secretions used by *S. carpocapsae* and *H. bacteriophora* against *O. nipae* immune system.

## Figures and Tables

**Table 1:** Prophenoloxidase activation genes Primers used for the qRT-PCR analysis.

Gene Name	Forward Primer (5′→3′)	Reverse Primer (5′→3′)
*qSPP56*	CGGTTGGTGGAAAGTGTCAG	CCCTCGTTGTCCAGCTTCTA
*qSPI28*	TCGCCTTAGTGATAGCGTGT	ACAGGGCTAGGGAAAACTCC
*qPPAF1*	GATCACCGGCGACAAAGAAA	CAGCTTGTTGGGATTGCCTT
*qPPO*	GTATCTTGTCACCCAATAGAGC	AAACGATTCAAGATGCCTGT
*q-RPS3-F*	GACGGTGTCTTCAAAGCTGA	ATTTCTGTACGTGTCGGGGT
